# Exploring the potential role of defensins in differential vector competence of body and head lice for *Bartonella quintana*

**DOI:** 10.1186/s13071-023-05802-4

**Published:** 2023-06-06

**Authors:** Kyungjae Andrew Yoon, Do Eun Lee, Si Hyeock Lee, Ju Hyeon Kim

**Affiliations:** 1 Research Institute of Agriculture and Life Sciences, Seoul National University, Seoul, 08826 Republic of Korea; 2 Department of Agricultural Biotechnology, Seoul National University, Seoul, 08826 Republic of Korea; 3 Department of Tropical Medicine and Parasitology, Seoul National University College of Medicine, Seoul, 03080 Republic of Korea; 4 Institute of Endemic Diseases, Seoul National University College of Medicine, Seoul, 03080 Republic of Korea

**Keywords:** Human louse, Antimicrobial peptide, Defensin, Antimicrobial activity, *Bartonella quintana*

## Abstract

**Background:**

The body and head lice of humans are conspecific, but only the body louse functions as a vector to transmit bacterial pathogens such as *Bartonella quintana*. Both louse subspecies have only two antimicrobial peptides, defensin 1 and defensin 2. Consequently, any differences in the molecular and functional properties of these two louse subspecies may be responsible for the differential vector competence between them.

**Methods:**

To elucidate the molecular basis of vector competence, we compared differences in the structural properties and transcription factor/microRNA binding sites of the two defensins in body and head lice. Antimicrobial activity spectra were also investigated using recombinant louse defensins expressed via baculovirus.

**Results:**

The full-length amino acid sequences of defensin 1 were identical in both subspecies, whereas the two amino acid residues in defensin 2 were different between the two subspecies. Recombinant louse defensins showed antimicrobial activities only against the representative Gram-positive *Staphylococcus aureus* but not against either Gram-negative *Escherichia coli* or the yeast *Candida albicans*. However, they did show considerable activity against *B. quintana*, with body louse defensin 2 being significantly less potent than head louse defensin 2. Regulatory sequence analysis revealed that the gene units of both defensin 1 and defensin 2 in body lice possess decreased numbers of transcription factor-binding sites but increased numbers of microRNA binding sites, suggesting relatively lower transcription activities of body louse defensins.

**Conclusions:**

The significantly lower antibacterial activities of defensin 2 along with the reduced probability of defensin expression in body lice likely contribute to the relaxed immune response to *B. quintana* proliferation and viability, resulting in higher vector competence of body lice compared to head lice.

**Graphical Abstract:**

**Figure:**
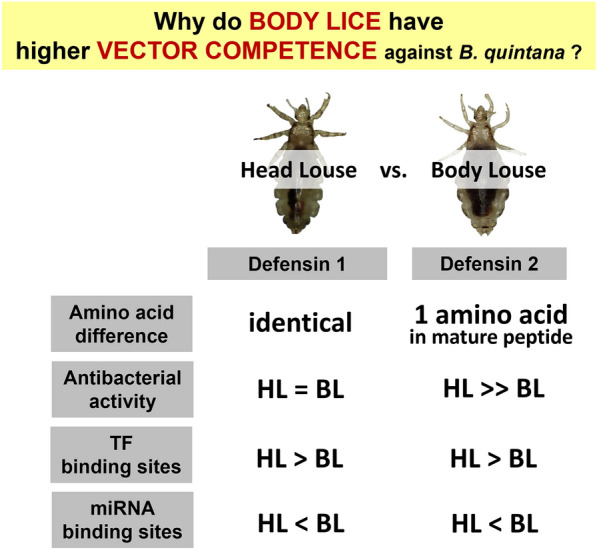

**Supplementary Information:**

The online version contains supplementary material available at 10.1186/s13071-023-05802-4.

## Background

The human body louse *Pediculus humanus humanus* and the head louse *P. h. capitis* are hematophagous ectoparasites that spend their entire life-cycle on human hosts and feed on human blood. Human head lice infestation causes economic and social problems, whereas the human body lice infestation threatens public health by vectoring bacterial diseases [1]. The body louse is believed to have evolved from a conspecific head louse 40,000–70,000 years ago, when humans began to wear clothing [2], and possesses higher vector competence than the head louse. Of these two lice types, only the body louse is known to transmit Gram-negative bacterial pathogens, including *Bartonella quintana, Rickettsia prowazekii* and *Borrelia recurrentis*, which cause trench fever, epidemic typhus, and relapsing fever, respectively. However, the head louse is not known to transmit bacterial diseases to humans, likely due to higher immune responses compared with those to the body louse [3–5].

A genome-wide analysis of representative immune-related genes in both body and head lice revealed a shared set of 93 genes, indicating a significantly shrunken immune system in human lice compared with other insects [3]. The number of genes associated with the humoral immune system is substantially reduced in human lice, with some immune genes absent in the human louse genome [3, 6]. This simplified immune system in human lice is suggested to be due to their parasitic life-cycle during which they feed exclusively on human blood, and blood is considered to be a relatively sterile diet. Interestingly, only two antimicrobial peptides (AMPs), namely defensin 1 and defensin 2, have been identified in the genomes of both body and head lice of humans [3]. Comparative transcriptome analysis revealed that both body and head lice have virtually the same genetic background [7], suggesting that the apparent differences in vector competence between these two types of lice may be due to the different transcriptional profiles of immune-related genes, such as the defensins. Basal transcription levels of both defensin 1 and defensin 2 were found to be significantly higher in the alimentary tract tissue of the head lice than in that of body lice [4]. In addition, defensin 1 expression could be induced in the alimentary tract tissues of the head louse but not in the body louse following an oral challenge with *B. quintana* [5]. Taken together, these findings suggest that the enhanced immune responses mediated by the constitutive and inducible expression of defensins 1 and 2 contribute to the reduced vector competence in the head louse.

Despite the importance of louse defensins in immune responses and vector competence in human lice, detailed information on the structural properties and functions of these AMPs is sparse. It remains to be elucidated how defensin expression is differentially regulated and whether there is any difference in the antimicrobial activity of defensins between the body and head lice. Furthermore, the efficacy of louse defensins against pathogenic bacteria such as *B. quintana* is yet to be determined.

In this study, we characterized the molecular and functional properties of louse defensins. The potential transcription factor-binding sites and microRNA (miRNA) binding sites for louse defensins were compared between body and head lice to elucidate their cross-subspecies differential expression profiles. Using in vitro expressed recombinant defensin 1 and defensin 2, the antimicrobial activity spectra of louse defensins were determined and compared between body and head lice. The data presented in this study should facilitate an in-depth understanding of the differential vector competence mediated by defensins 1 and 2 between body and head lice.

## Methods

### Lice rearing

The San Francisco strain of body louse and the South Florida strain of head louse were reared in an in vitro membrane feeding system [8] under controlled conditions of 30 °C, 70–80% relative humidity and 16/8-h light/dark in rearing chambers (Institutional Review Board No. E2211/001–003).

### Sequence analysis

Defensin complementary DNA (cDNA) sequences from body and head lice were obtained from the National Center for Biotechnology Information (NCBI; https://www.ncbi.nlm.nih.gov/) and a previous publication [6], and confirmed by the cloning and resequencing of cDNA from the San Francisco strain of body louse and the South Florida strain of head louse. Amino acid sequences of defensins from 38 insect species were aligned using the CLC Main Workbench 8 analysis package (CLC Bio, Waltham, MA, USA). A phylogenetic tree was constructed using MEGA X (Pennsylvania State University, University Park, PA, USA) using the maximum likelihood method based on the Jones-Taylor-Thornton (JTT) model with 1000 bootstrap replications. The number on each node of the tree indicates the percentage of bootstrap values based on 1000 pseudo-replicates. Three-dimensional (3D) structural modeling of defensins was conducted based on the structures of insect defensin A [9] and *Anopheles* defensin [10] peptides in the Protein Data Bank. The sequences of the louse genes were submitted to the molecular modeling server of the SWISS-MODEL (Automated Comparative Protein Modeling Server) [11] for structure prediction, and 3D structures were analyzed using the UCSF Chimera program [12]. The signal peptide cleavage site, hydrophobicity, molecular weight, isoelectric point (pI), and net charge at pH 7 were predicted using the SignalP 6.0 server (https://services.healthtech.dtu.dk/service.php?SignalP) [13], ProtScale tool (https://web.expasy.org/protscale/) [14], Compute pI/MW tool (https://web.expasy.org/compute_pi/) [14] and peptide property calculator (https://pepcalc.com/), respectively. Transcription factor-binding sites in the 5’ upstream region of genes were predicted using the motif discovery program PROMO (http://alggen.lsi.upc.es/). The 1000-bp putative regulatory region of the target gene (800-bp upstream plus 200-bp downstream genomic DNA sequences from the transcription starting site) was used for transcription factor-binding site prediction. The sequences were obtained from the vector base (http://www.vectorbase.org) and head louse genome sequencing data [6] for body and head lice, respectively. The 3’-untranslated regions (UTRs) in defensins 1 and 2 transcripts from the body and head lice were used for miRNA prediction using the miRNA sequence database miRBase (http://mirbase.org) [15]. The E-value cutoff was 10, and the arthropod miRNA sequence database was used for specific predictions.

### Generation of recombinant human louse defensins via a baculovirus expression system

Total RNA from five body and head lice was extracted using 200 μl of TRI reagent (Molecular Research Center, Cincinnati, OH, USA), according to the manufacturer’s instructions. To remove DNA contamination from total RNA, DNase I (Takara Bio, Kyoto, Japan) was treated and cDNA was synthesized using SuperScript IV reverse transcriptase (Invitrogen, Thermo Fisher Scientific, Waltham, MA, USA) with oligo dT primers. The signal peptide and mature peptide regions of defensins 1 and 2 of the body louse and defensin 2 of the head louse were separately amplified using gene-specific primers (Additional file 1: Table S1) with Advantage Taq (Clontech, Palo Alto, CA, USA). These were then inserted together into the pBacPAK8 vector (Clontech) with complementary single-stranded primers containing a 6× His-Tag. The plasmid sequences for each cloning step were verified by DNA sequencing (Macrogen, Seoul, Korea). Transfer vectors containing defensins were co-transfected with BacPAK6 DNA (Clontech) into Sf9 cells (Invitrogen, Thermo Fisher Scientific) cultured in TC-100 medium (WelGENE, Gyeongsan, Korea) supplemented with 10% fetal bovine serum (WelGENE) at 27 °C (Additional file 2: Table S2). After 5 days of transfection with Bacfectin reagent (Clontech), the supernatant was collected from the infected cells and used as a virus stock. The first virus stock was seeded on 5 × 10^6^ Sf9 cells, and the supernatant was collected after 3 days of infection via centrifugation at 3300 rpm for 15 min at 4 °C. The supernatant was concentrated using an Amicon Ultra-4 Centrifugal Filter Unit-3 kDa (MilliporeSigma, Burlington, MA, USA) by centrifugation at 7500 × *g* for 40 min at 4 °C. The concentrated supernatant was loaded into a 5-ml HisTrap HP affinity column (GE Healthcare, Pittsburgh, PA, USA) and equilibrated with a binding buffer (50 mM NaH_2_PO_4_ and 300 mM NaCl containing 5 mM imidazole, pH 8.0) at a flow rate of 5 ml/min using AKTAprime plus FPLC (GE Healthcare). The 5-ml HisTrap HP affinity column was washed with 40 ml of washing buffer (50 mM NaH_2_PO_4_ and 300 mM NaCl containing 10 mM imidazole, pH 8.0) and eluted with elution buffer (50 mM NaH_2_PO_4_ and 300 mM NaCl containing 250 mM imidazole, pH 8.0). Fractions with elution peaks were collected and concentrated using an Amicon Ultra-4 Centrifugal Filter Unit-3 kDa (MilliporeSigma) via centrifugation at 7500 ×* g* for 40 min at 4 °C, and the buffer was exchanged with 100 mM Tris–HCl (20 mM NaCl, pH 7.8) buffer to reduce the imidazole concentration. The concentration of the purified peptide sample was quantified using the Pierce BCA Protein Assay Kit (Invitrogen, Thermo Fisher Scientific), with bovine serum albumin as a standard protein.

### Antimicrobial activity assay

Gram-negative *Escherichia coli* (American Type Culture Collection [ATCC] no. 11775), Gram-positive *Staphylococcus aureus* (ATCC no. 12600) and Gram-positive yeast *Candida albicans* (ATCC no. 10231) were cultured in Luria–Bertani broth, brain heart infusion broth and potato dextrose broth, respectively, at 200 rpm in a shaking incubator at 37 °C overnight, following which the cultured bacteria were diluted by 100-fold in the same culture broth. After the optical density reached 0.5 at 600 nm (OD_600_), 10 μl aliquots of each of recombinant defensins 1 and 2 at various concentrations were each incubated with 90 μl of bacterial culture in a 96-well plate in a 37 °C shaking incubator at 200 rpm overnight. Tris-HCl buffer was used as the negative control. The OD_600_ values were measured using a VersaMax microplate reader (Molecular Devices, San Jose, CA, USA). The half-maximal inhibitory concentration (IC_50_) was calculated using GraphPad Prism 6 (GraphPad Software, San Diego, CA, USA), and antimicrobial activity assays were conducted with three replications.

### *Bartonella quintana* culture and inhibition assay

The *B. quintana* JK31 wild-type strain, originally obtained from Dr. Jane Koehler (University of California, San Francisco, CA, USA), was maintained in a biosafety level 2 facility at Seoul National University (LML08-1090). Frozen stocks of *B. quintana* were cultured on a chocolate agar plate in a candle extinction jar at 37 °C for 10 days and passed to a fresh agar plate for an additional 7 days of culture. Cultured *B. quintana* cells were harvested and rinsed with 1 ml of phosphate-buffered saline (PBS) by centrifugation at 1000 × *g* for 4 min. Following two additional rinses, the pellets were re-suspended in 100 μl of PBS. Aliquots of 5 μl of the 100 μl bacterial suspension were serially diluted with PBS; then 12 μl of each 1000-fold diluted bacterial suspension was thoroughly mixed with 12 μl of 100 μM gentamicin as a positive control, 100 mM Tris–HCl buffer as a negative control and 100 μM recombinant defensins 1 and 2 from the body louse or defensin 2 from the head louse. A total of 8 μl of the mixture was dropped onto a chocolate agar plate three times, and the number of colony-forming units (CFUs) was calculated after incubation in a candle extinction jar at 37 °C for 10 days. The colony index was obtained by dividing CFUs/μl by CFUs/μl of the negative control.

### Hemolytic activity assay

The hemolytic activities of recombinant defensins 1 and 2 from the body louse and defensin 2 from the head louse were evaluated using human red blood cells (RBCs) as previously described [16]. RBCs were washed 3 times with PBS for 15 min at 960 rpm and then re-suspended in PBS at a concentration of 2%. Aliquots of 10 μl of recombinant defensins at four concentrations (10, 20, 50 and 100 μM) and antibiotics at five concentrations (10, 20, 50, 100 and 200 μM) were each incubated with 90 μl of RBCs for 30 min at 37 °C and centrifuged for 15 min at 960 rpm. The OD_540_ of the supernatant was measured using a VersaMax microplate reader (Molecular Devices), and the relative hemolytic activities of Tris–HCl buffer and 0.1% Triton X-100 were considered to be 0% and 100%, respectively. Hemolytic assays were conducted with three replications, and the protocol was approved by the Institutional Review Board (Institutional Review Board No. E2211/001–003).

### Statistical analysis

At each time point, all experimental data were collected in triplicate. One-way analysis of variance with Tukey’s multiple comparison test and unpaired t-tests were used to determine significant differences by interpreting *P*-values. Statistical significance was set at *P* < 0.05, and statistical analysis was performed using the GraphPad Prism 6.01 software (GraphPad Software).

## Results

### Amino acid alignments and phylogenetic analysis of defensins 1 and 2 of body and head lice

**Fig. 1:**
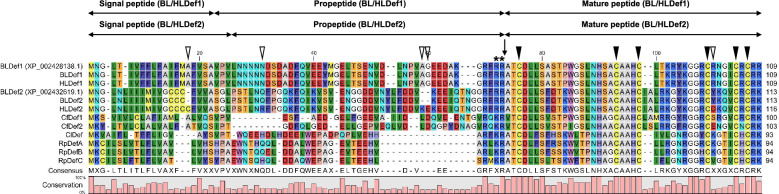
Alignments of the amino acid sequences of defensin 1 and defensin 2 of body and head lice along with other blood-feeding insects, including the cat flea and hemipteran species. Arrow indicates the start point of mature peptide regions, asterisks indicate the dipeptide cleavage site for trypsin-like proteases, open triangles indicate different amino acids between BLDef2 and HLDef2, filled triangles indicate the conserved six cysteine residues. The sequences of BLDef1 (XP_002428138.1) and BLDef2 (XP_002432619.1) were obtained from the National Center for Biotechnology Information (NCBI) database. BL, body louse; Def1, defensin 1; Def2, defensin 2; Cf, *Ctenocephalides felis*; Cl, *Cimex lectularius*; HL, head louse; Rp, *Rhodnius prolixus*

Defensins 1 and 2 of the body and head lice were aligned using the CLC Main Workbench 8 (Qiagen, Hilden, Germany) (Fig. 1). The open reading frame (ORF) sequences of body louse defensin 1 (BLDef1) and head louse defensin 1 (HLDef1) matched completely with the reference BLDef1 sequence (Accession Number: XP_002428138.1). BLDef1 and HLDef1 were identical and consisted of 109 amino acids, including a 20-amino acid signal peptide, a 46-amino acid propeptide and a 43-amino acid mature peptide region. The ORF sequence of body louse defensin 2 (BLDef2) was completely identical to the reference sequence (Accession Number: XP_002432619.1) but was slightly different from the head louse defensin 2 (HLDef2) sequence. Compared to the 116-amino acid HLDef2 sequence, BLDef2 was composed of 113 amino acids due to the deletion of one cysteine residue in the signal peptide region and two residues (lysine and glutamic acid) in the propeptide region; in addition, two amino acid substitutions were found between BLDef2 and HLDef2: one in the propeptide region (glutamine at amino acid position 29 for BLDef2 vs. arginine at amino acid position 30 for HLDef2) and the other in the mature peptide region (tyrosine at amino acid position 105 for BLDef2 and aspartic acid at amino acid position 108 for HLDef2). The radical amino acid replacement of aspartic acid with tyrosine in the mature peptide of BLDef2 implied the possibility of different biological activities between BLDef2 and HLDef2.

Conserved dipeptide cleavage sites (-RR- and -KR- in defensins of the human louse and other insects, respectively) for trypsin-like proteases were found between the propeptide and mature peptide regions [17], and six conserved cysteine residues were present in the mature peptide regions of all the defensins examined (Fig. 1). The start point of mature peptide regions in all the defensins contained alanine and threonine residues (-AT-), with the exception of defensin 1 and 2 of cat flea (-VT-). The C-terminal region of all the examined defensins showed a high conservation of two basic residues, either arginine-arginine (RR) or arginine-lysine (RK). The sequence similarities of mature peptides in defensins 1 and 2 across the examined insect species were relatively higher (51.2–72.1% and 60.5–66.7%, respectively) compared to the full-length sequences (33.3–46% and 31.9–35.9%, respectively).

**Fig. 2:**
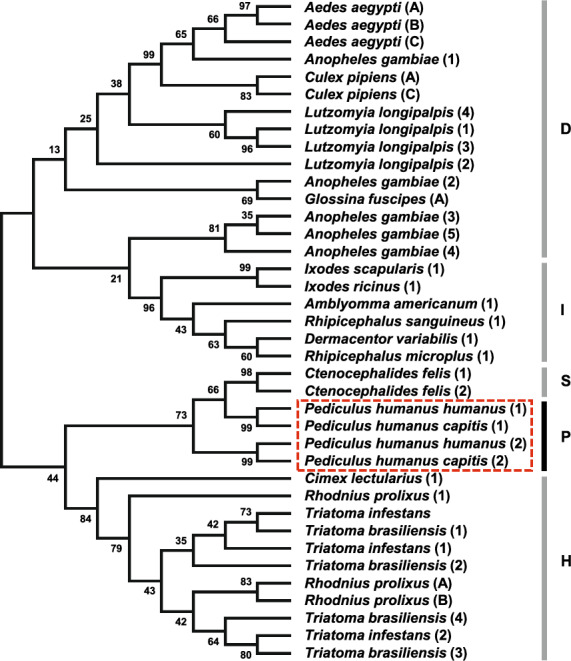
Phylogenetic tree of defensins from insect species. The red-dotted box indicates the clade of louse defensins. Phylogenetic analysis was conducted using the maximum likelihood method based on the Jones-Taylor-Thornton (JTT) model with 1000 bootstrap replications. The percentages of bootstrap values are represented on each node of the tree. D, Diptera; I, Ixodida; S, Siphonaptera; P, Phthiraptera; H, Hemiptera

Defensins 1 and 2 of 33 insect species (2 Phthiraptera, 6 Diptera, 14 Hymenoptera, 5 Hemiptera, 3 Coleoptera, 1 Siphonaptera, 1 Thysanoptera, 1 Orthoptera) were aligned with those of body lice, and defensins 1 and 2 of 46 insect species (2 Phthiraptera, 5 Diptera, 30 Hymenoptera, 4 Hemiptera, 2 Coleoptera, 2 Lepidoptera, 1 Siphonaptera) were aligned with those of head lice (Additional file 3: Figure S1). The mature peptide regions of the defensins of insect species showed higher sequence similarities than those of the 5’ sequences. In particular, six cysteine residues were conserved in most mature peptide regions, demonstrating that the six cysteine residues are one of the representative structural motifs in most insect defensins. In the phylogenetic tree, defensin 1 and 2 of body and head lice clustered with defensins 1 and 2 of the cat flea, *Ctenocephalides felis* (Siphonaptera), despite the taxonomic distance between lice and fleas (Fig. 2).

### Structural properties of defensins 1 and 2 of body and head louse

BLDef1 and HLDef1 possessed the least number of positively charged amino acids (14), thus showing a relatively lower pI value (6.84) and net charge at pH 7 (- 0.2). The hydrophobicity (40.37%) of BLDef1 and HLDef1 was higher than that of BLDef2 and HLDef2 (35.34%) (Table 1). The overall structural properties of BLDef2 and HLDef2, such as the pI value, net charge and hydrophobicity, were identical except for the number of positively charged amino acids (18 and 19, respectively). BLDef1 and HLDef1 also exhibited the lowest number of positively charged amino acids (10) among the propeptide-removed defensins but showed the highest pI (9.61) and net charge (6.8). Propeptide-removed BLDef2 and HLDef2 possessed identical numbers of positively charged amino acids (11) and identical percentage of hydrophobicity (38.24%), whereas the pI (8.46 and 8.29, respectively) and net charge (6.5 and 5.5, respectively) were different and lower than those of propeptide-removed BLDef1 and HLDef1. Native defensins and propeptide-removed defensins from the body and head lice exhibited similar molecular weights (12.1–12.9 and 6.9–7.4 kDa, respectively).

**Table 1:** Structural properties of defensins 1 and 2 of body louse

Antimicrobial peptides	Amino acid sequences	Number of positively charged amino acid residues	Hydrophobicity (%)	Molecular weight (g/mol)	Iso-electric point	Net charge at pH 7
BLDef1 & HLDef1	MNGLTIVFFLFAIFMAFVSAVPVLNNNNNDSDADFQVEEYMGELTSENVDLNPVAGEEDAKGRFRRATCDLLSASTPWGSLNHSACAAHCLTKRYKGGRCRNGICRCRR	14	40.37	12,068.57	6.84	−0.2
BLDef2	MNGLNLIIIMIVGCCCCFVVASGLPSTLNQFPGQKFQIKVSVENGGDDVNYLFDDVKEKEEIQTNGGRFRRATCDLLSFDTKWGSLNHSACAAHCIALRKGYKGGRCYKQVCRCRK	18	35.34	12,873.88	8.28	5.5
HLDef2	MNGLNLIIIMIVGCCCCFVVASGLPSTLNRFPGQKFQIKVSVENGGDDVNYLFDDVKEKEEIQTNGGRFRRATCDLLSFDTKWGSLNHSACAAHCIALRKGYKGGRCDKQVCRCRK	19	35.34	12,853.85	8.28	5.5
BLDef1 & HLDef1 (pro-peptide removed)	MNGLTIVFFLFAIFMAFVSAATCDLLSASTPWGSLNHSACAAH CLTKRYKGGRCRNGICRCRR	10	44.44	6917.15	9.61	6.8
BLDef2 (pro-peptide removed)	MNGLNLIIIMIVGCCCCFVVASGATCDLLSFDTKWGSLNHSACAAHCIALRKGYKGGRCYKQVCRCRK	11	38.24	7376.87	8.46	6.5
HLDef2 (pro-peptide removed)	MNGLNLIIIMIVGCCCCFVVASGATCDLLSFDTKWGSLNHSACAAHCIALRKGYKGGRCDKQVCRCRK	11	38.24	7328.78	8.29	5.5

*BLDef1 & HLDef1* Body louse & head louse defensin 1,* BLDef2* body louse defensin 2, HLDef2 head louse defensin 2

**Fig. 3:**
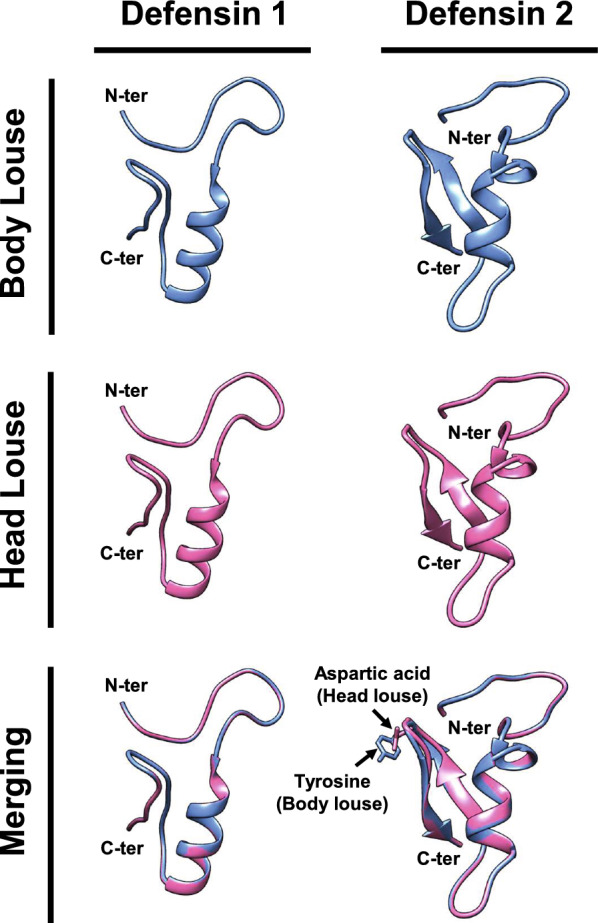
Three-dimensional protein structures of louse defensin 1 (left panel) and defensin 2 (right panel) from body (top) and head lice (middle). Bottom image is the merged image. Side chains of different amino acids between body louse defensin 2 and head louse defensin 2 are shown. N-ter, N-terminal; C-ter, C-terminal

A 3D structural analysis revealed that BLDef1 and HLDef1 both consisted of a single, identical α-helix (His83–Lys93), whereas BLDef2 and HLDef2 comprised one α-helix (Asn87-Ile96) and two β-sheets (β1: Gly104-Cys107; β2: Cys112-Arg115) (Fig. 3). The mature peptide of defensin 2 contained one different amino acid in the loop between the two antiparallel β-sheets, which caused a slight distortion of the loop, as noted from examination of the merged image (Fig. 3); however, the overall structures of BLDef2 and HLDef2 were almost identical.

### Regulatory sequence properties of defensins 1 and 2 of body and head lice

**Fig. 4:**
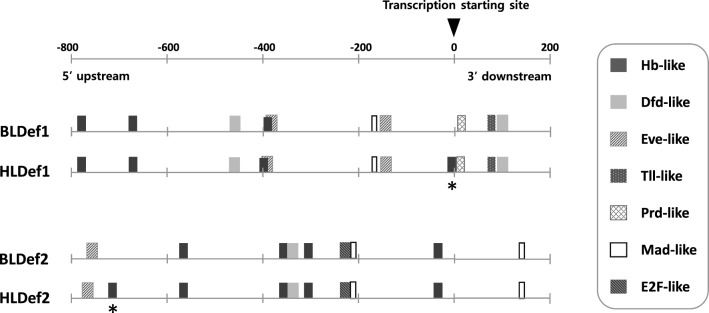
Potential transcription factor-binding motifs observed in the putative regulatory region (800 bp upstream and 200 bp downstream from the gene transcription start site). Bars with different colors and patterns represent different potential motifs. Different motifs between the body and head louse are denoted with an asterisk. BLDef, Body louse defensin; HLDef, head louse defensin; Dfd, deformed; Eve, even skipped; Hb, hunchback; Mad, mothers against dpp; Prd, paired; T11, tailless

Six putative transcription factor-binding sites, including hunchback (Hb)-like, deformed (Dfd)-like, even skipped (Eve)-like, tailless (Tll)-like, paired (Prd)-like and mothers against dpp (Mad)-like sites, were commonly identified in the putative regulatory regions (800 bp upstream and 200 bp downstream from the transcription start site) of *BLDef1* and *HLDef1*, with one additional Hb-like binding site being present at 796–802 bp upstream of *HLDef1* (Fig. 4). In the case of defensin 2, five transcription factors, including Hb-like, Dfd-like, Eve-like, Mad-like, and e2 factor (E2F)-like sites, were commonly found in both *BLDef2* and *HLDef1*, with an additional Hb-like site being present at 88–94 bp upstream of *HLDef2* (Fig. 4).

Six putative miRNA-binding sites were predicted in the 3′UTR of the *BLDef1* transcript, whereas three putative miRNA-binding sites were predicted in the *HLDef1* transcript (Table 2). In the 3’UTR of defensin* 2* transcripts, five putative miRNA-binding sites were predicted in *BLDef2,* whereas four putative miRNA-binding sites were predicted in *HLDef2* (Table 2).

**Table 2:** Predicted microRNA binding sites in 3’ untranslated region sequences of messenger RNA from defensin 1 and defensin 2 of body and head lice

mRNAs	miRNAs	Species	Sequences	Query start	Query end	E-value	Body louse^a^	Head louse^a^
Defensin 1	*miR-11654-3p*	*Dinoponera quadriceps*	aaauuucaaauuuuuuuuuuca	9	30	2.7	O	X
	*miR-8519*	*Plutella xylostella*	uuuucaaaucauuacauuu	25 (BL) 24 (HL)	43 (BL) 42 (HL)	0.47 (BL) 0.47 (HL)	O	O
	*miR-10358-3p*	*Anopheles gambiae*	uuucauuuuauuuuacuauuaaauuuu	90	116	6.9	O	X
	*miR-972-5p*	*Drosophila melanogaster*	uaaauuuuuuuuuuuuuu (BL) uaaauuuuuuuuuug (HL)	109 (BL) 132 (HL)	126 (BL) 146 (HL)	6.9 (BL) 3.9 (HL)	O	O
	*miR-3853-5p*	*Tribolium castaneum*	uauauuuuguuaaauguua	138	156	2.7	O	X
	*miR-11900*	*Aedes aegypti*	auuuuguuaaauguuauuuua	141	161	2.2	O	X
	*miR-3429*	*Bombyx mori*	uuaauaauuucuu	106	118	4.7	X	O
Defensin 2	*miR-10404-3p*	*Drosophila melanogaster*	ugaaaaaaaaaaaaaaaaca	2, 101	21, 122	8.3, 6.8	O	X
	*miR-2551-5p*	*Drosophila pseudoobscura*	aaaaaaaaaaaacaauuuaauuu	8 (BL) 9 (HL)	30 (BL) 31 (HL)	2.6 (BL) 2.4 (HL)	O	O
	*miR-3837-5p*	*Tribolium castaneum*	auuuaauuauuagucguuuguu	27 (BL) 28, 89 (HL)	48 (BL) 49, 106 (HL)	6.8 (BL) 6.3, 7.6 (HL)	O	O
	*miR-6038-5p*	*Apis mellifera*	uuuguuucugucuuauuu	43 (BL) 44 (HL)	60 (BL) 61 (HL)	0.23 (BL) 0.29 (HL)	O	O
	*miR-11900*	*Aedes aegypti*	uauuaaauuaaaagaaaaaaaa	95	116	6.8	O	X
	*miR-2998*	*Bombyx mori*	uuauuuaccucgucuuguuuu	56	76	2.9	X	O

*BL* Body louse, *HL* Head louse,* miRNA* microRNA,* mRNA* messenger RNA

^a^Identified or non-identified miRNAs in defensin 1 and defensin 2 of body and head lice were marked as “O” or “X”, respectively

### Antimicrobial activities of recombinant defensins of body and head lice

The positive controls, including ampicillin, kanamycin and gentamicin, inhibited the growth of *E. coli* and *S. aureus*; however, the growth of *C. albicans* was not inhibited, even at the highest concentrations of these antibiotics (200 μM) and recombinant BLDef1 and BLDef2 (100 μM) (Table 3). Ampicillin inhibited *S. aureus*, with 44.5-fold lower half-maximal inhibitory concentration (IC_50_) values (*P* = 0.0007) than those against *E. coli*, whereas kanamycin inhibited *E. coli*, with 8.5-fold lower IC_50_ values (*P* = 0.002) than those against *S. aureus,* indicating that these two antibiotics possess highly specific antimicrobial activities that depend on the bacterial species. Gentamicin exhibited lower IC_50_ values against *E. coli* and *S. aureus* than the other two antibiotics and recombinant defensins of the body and head lice. Purified recombinant BL/HLdef1 and BLDef2 only inhibited the growth of *S. aureus*, with similar IC_50_ values (33.2 ± 1.4 μM and 34.4 ± 0.8 μM, respectively; *P* = 0.2669), which were about 20- and 48-fold less potent than ampicillin (*P* < 0.0001) and gentamicin (*P* < 0.0001), respectively, but 1.8-fold more potent than kanamycin (*P* = 0.0093 and 0.0114, respectively). HLDef2 showed 6.1-, 6.4- and 11.4-fold lower IC_50_ values against *S. aureus* than BL/HLDef1 (*P* = 0.004), BLDef2 (*P* = 0.0031) and kanamycin (*P* < 0.0001), respectively. These results indicate that louse defensins possess more effective Gram-positive bacteria-specific antimicrobial activity than kanamycin, and that HLDef2 possesses significantly higher antimicrobial activity against Gram-positive *S. aureus* than BLDef2.

**Table 3:** Antimicrobial activities of recombinant defensins from body and head lice and antibiotics against *Escherichia coli*, *Staphycoccus aureus* and *Candida albicans*

Antimicrobial peptides	Antimicrobial activity (IC_50_, μM)		
	*Escherichia coli*	*Staphylococcus aureus*	*Candida albicans*
Ampicillin	75.7 ± 13.6	1.7 ± 0.5	NI
Gentamicin	0.6 ± 0.1	0.7 ± 0.02	NI
Kanamycin	7.3 ± 0.3	61.7 ± 13.2	NI
BL/HLDef1	NI	33.2 ± 1.4	NI
BLDef2	NI	34.4 ± 0.8	NI
HLDef2	NI	5.4 ± 0.1	NI

*BL/HLDef1* Body louse/head louse defensin 1,* BLDef2* body louse defensin 2, * HLDef2* head louse defensin 2,* IC*_*50*_ half-maximal inhibitory concentration, *NI* no detectable inhibition

**Fig. 5:**
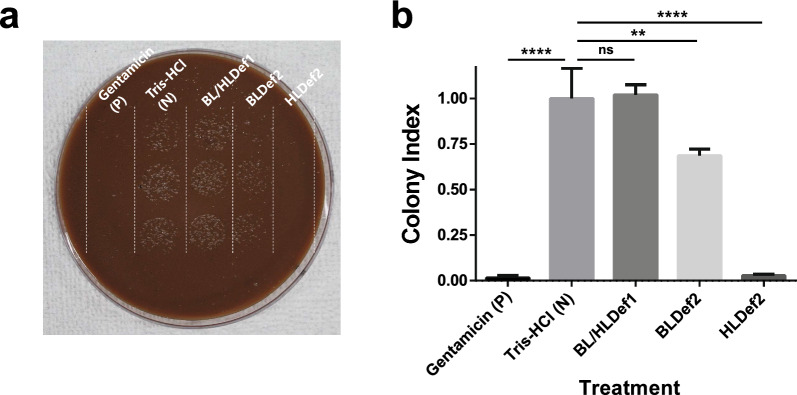
**a** Representative image of gentamicin (P, positive control), Tris–HCl buffer (N, negative control) and BL/HLDef1-, BLDef2- or HLDef2-treated *Bartonella quintana* mixture dropped onto chocolate agar plate. Gentamicin and Tris–HCl buffer were used as the positive and negative control, respectively. **b** Result of the inhibition assay of the *B. quintana* mixture with recombinant louse defensins. Colony index was obtained from colony-forming units/microliter (CFUs/μl) divided by CFUs/μl of negative control under the assumption that *B. quintana* is not inhibited by Tris–HCl buffer. Results were statistically analyzed using one-way analysis of variance Asterisks indicate significant differences at **P < 0.01 and ***P < 0.0001; ns, no significant difference. BL/HLDef1, Body louse/head louse defensin 1; BLDef2, body louse defensin 2; HLDef2, head louse defensin 2

Treatment with 50 μM gentamicin as a positive control resulted in a 98.4% inhibition of *B. quintana* colony growth compared with the Tris–HCl buffer-treated negative control (Fig. 5). Recombinant HLDef2 exhibited significantly higher antibacterial activity against *B. quintana* (97.2%) (*P* < 0.0001) than BLDef2 (31.5%) (*P* = 0.0023). Recombinant BL/HLDef1 did not exhibit any apparent antibacterial activity against *B. quintana*.

## Discussion

Insect defensins are generally active against a broad spectrum of pathogens [18]. Although recombinant BL/HLDef1 was only active against Gram-positive *S. aureus*, recombinant BL/HLDef2 exhibited significantly high antibacterial activity against both Gram-positive *S. aureus* and Gram-negative *B. quintana*. Similar cases, in which defensin showed activity against Gram-negative bacteria, have been previously reported in a variety of arthropods [19–24]. However, louse defensins did not show any apparent hemolytic activity despite their substantially high antibacterial activities, an observation also reported for other defensins [17, 25].

Insect defensins typically have an N-terminal loop and an *α*-helix followed by an antiparallel β-sheet structure connected by disulfide bonds [26]. BL/HLDef2 showed these unique structures; however, BL/HLDef1 seemed to have only an *α*-helical fragment and three defensin-specific cysteine pairs without an β-sheet. In addition, louse defensins showed high pI values, similar to other insect defensins that exert antibacterial activity through the interaction between positively charged peptides and negatively charged bacterial membrane components (i.e. polysaccharide and lipopolysaccharide in Gram-positive and Gram-negative bacteria, respectively) [27].

Among the three domains of defensins (signal peptide, propeptide and functional mature peptides), the propeptide is generally considered to inhibit the antimicrobial activity of the C-terminal domain in human neutrophil *α*-defensins (HNPs) by its opposite net charges [28]. Activation of cationic HNPs requires proteolytic excision of the anionic propeptide [29], indicating that electrostatic forces might be critical for inhibitory intermolecular interactions between the cationic mature peptide and anionic propeptide of defensin [29]. The propeptides and mature peptides of both BL/HLDef1 and BL/HLDef2 also exhibited opposite net charges: the net charge of the cationic mature peptide of BL/HLDef1 was 7.8, and those of BLDef2 and HLDef2 were 7.8 and 6.8, respectively. In contrast, the net charges of the anionic propeptide of BL/HLDef1 were - 8, and those of BL/HLDef2 were - 2 and - 1, respectively. Since these findings indicate a potential inhibitory electrostatic interaction between the propeptide and mature peptide of louse defensins, we expressed only the mature peptide domains of louse defensins without propeptides and investigated their biological properties.

Recombinant HLDef2 showed significantly higher antibacterial activity against Gram-negative *B. quintana* and Gram-positive *S. aureus* than either recombinant BL/HLDef1 or BLDef2. Since only one amino acid residue (tyrosine for BLDef2 vs. aspartic acid for HLDef2) in the mature peptide region of defensin 2 differs between body and head lice, this amino acid difference is likely responsible for the differential antibacterial activity against *B. quintana* and *S. aureus*. The only significantly different factor caused by the amino acid substitution in structural properties between BLDef2 and HLDef2 was net charge at pH 7. The net charge increase (+ 6.5 for BLDef2 vs. + 5.5 for HLDef2) resulted from the substitution of aspartic acid with tyrosine in BLDef2 and may contribute to the reduction in the antibacterial activity against Gram-negative *B. quintana* and Gram-positive *S. aureus,* as generally observed in insect defensins, where lower net charge is associated with high antimicrobial activities against Gram-negative bacteria [30]. Furthermore, as demonstrated by the engineering of the *Nasonia vitripennis* defensin as a model [31], the β-sheet subdomain of insect defensins is an important factor for broad antibacterial activity against Gram-positive and Gram-negative bacteria. Therefore, the structural alteration in the β-sheet subdomain caused by substitution with a tyrosine residue at the β-turn is likely to affect the antibacterial activity of BLDef2. Taken together, the increased net charge and structural change in the β-sheet subdomain of BLDef2 appear to be the primary determinants of the reduced antibacterial activity, particularly against *B. quintana* in body lice, which in turn has likely increased vector competence.

The only difference in the transcription factor binding sites of defensin 1 and 2 between the body and head lice was the presence of an additional Hb-like protein binding site in the regulatory regions of *HLDef1* and *HLDef2*. Hunchback belongs to the C_2_H_2_ zinc finger protein family that functions as a gene regulatory protein [32]. It is unclear whether Hb-like proteins activate or repress the transcription of *defensin* genes in the body and head lice. Nevertheless, assuming the transcription-activating role of Hb-like protein in lice, the presence of an additional binding site for Hb-like protein in either *HLDef1* or *HLDef2* may result in a higher basal transcription level in head lice [4] and in the inducible expression of defensin 1 in the alimentary tract tissues of head lice following oral challenge with *B. quintana* [5]. miRNAs are a class of short, endogenous and non-coding RNAs with a length of 21–24 nucleotides that regulate gene expression via base pairing with mRNAs [33]. The “seed sequence” of miRNA with two to eight nucleotides at the 5’ end binds to the complementary match site in the 3’UTR of mRNAs, resulting in the inhibition of translation and expression of the target gene or mRNA degradation [34]. An increased number of putative miRNA binding sites (6) was predicted in the 3’UTR of *BLDef1* transcript compared to *HLDef1* transcript (3). Likewise, an increased number of putative miRNA binding sites (5) was predicted in the 3’UTR of *BLDef2* compared to the *HLDef2* transcript (4). These findings suggest that the expression of both *BLDef1* and *BLDef2* is more likely to be downregulated than that of *HLDef1* and *HLDef2* in head louse. Therefore, the relatively reduced expression of BLDef1 and BLDef2 in the body lice appears to contribute to enhanced vector competence [5].

Understanding the immune system of a human disease-transmitting vector is of great importance because it is the immune system that largely determines vector competence. However, the immune defense cascade against bacterial pathogens, such as *B. quintana*, *R. prowazekii* and *B. recurrentis*, in body lice still remains largely unknown. Nevertheless, we elucidated (i) that body lice have a functionally altered defensin 2, which is significantly less effective against *B. quintana*, a model pathogen used in this study, and (ii) that the expression of both defensin 1 and defensin 2 is likely downregulated due to the difference in the regulatory sequences for transcription factor binding and miRNA binding. Therefore, the relatively higher vector competence of body lice appears to be mainly due to the reduced antibacterial activity of defensin 2 against pathogenic bacteria and the lower amounts of both BLDef1 and BLDef2.

## Conclusions

Defensin 1 and defensin 2, the only antimicrobial peptides in human body and head lice, exhibit differences in structure and antimicrobial activity. The significantly lower antibacterial activities of defensin 2 along with the reduced probability of defensin expression and transcription activities in body lice likely contribute to the relaxed immune response to *B. quintana* proliferation and viability, resulting in higher vector competence of body lice compared to head lice. These data will facilitate an in-depth understanding of the differential vector competence mediated by defensins 1 and 2 between body and head lice.

## Supplementary Information

**Additional file 1: Table S1.** Primers used for in vitro expression of defensin 1 and 2 of body and head louses.

**Additional file 2: Table S2.** Hemolytic activities of recombinant defensins from body and head lice and antibiotics.

**Additional file 3: Figure. S1.** Amino acid sequence alignments of defensin 1 and 2 from body and head louse and other insect species. Six conserved cysteine residues are marked by red boxes.

## Data Availability

Not applicable.

## Abbreviations

AMP: Antimicrobial peptide
BLDef: Body louse defensin
CFU: Colony-forming unit
Dfd: Deformed
E2F: E2 factor
Eve: Even skipped
Hb: Hunchback
HLDef: Head louse defensin
IC_50_: Half-maximal inhibitory concentration
Mad: Mothers against dpp
miRNA: MicroRNA
PBS: Phosphate-buffered saline
Prd: Paired
RBC: Red blood cell
Tll: Tailless
