# Supplementary material for: Exploring the potential role of defensins in differential vector competence of body and head lice for Bartonella quintana
Source: Parasit Vectors. 2023 Jun 6;16:183. doi: 10.1186/s13071-023-05802-4 (PMC10243063; doi:10.1186/s13071-023-05802-4)
Supplement: Supplementary file 1 — Additional file 1: Table S1. Primers used for in vitro expression of defensin 1 and 2 of body and head louses. [file 13071_2023_5802_MOESM1_ESM.docx]

**Table S1.** Primers used for *in vitro* expression of defensin 1 and 2 of body and head louses.

| **Name** | **Sequence** | **Length (bp)** | **Tm (℃)** | **%GC** |
| --- | --- | --- | --- | --- |
| BLDef1-signal-F-XbaI | GAATCTAGAATGAACGGATTAACGATTGTTT | 22 | 54.7 | 32 |
| BLDef1-signal-R-SacI | CTTGAGCTCGGCAGAAACAAAAGCCATAA | 20 | 54.3 | 40 |
| BLDef1-mature-F-SacI | GAAGAGCTCAGAGCAACATGTGACTTATTG | 21 | 55.4 | 38 |
| BLDef1-mature-R-KpnI | CTTGGTACCTCTTCTGCATCTGCAAATTC | 20 | 54.3 | 40 |
| BLDef2-signal-F-XbaI | GAATCTAGAATGAACGGTTTGAATTTGA | 25 | 59.2 | 32 |
| BLDef2-signal-R-BglII | CTTAGATCTACCTGATGCCACGACGAAG | 19 | 59.5 | 58 |
| BLDef2-mature-F-BglII | GAAAGATCTAGAGCAACTTGTGATCTATTGAG | 23 | 59.2 | 39 |
| BLDef2-mature-R-KpnI | CTTGGTACCTTTTCGACAGCGG | 19 | 59.5 | 58 |
