# Supplementary material for: Exploring the potential role of defensins in differential vector competence of body and head lice for Bartonella quintana
Source: Parasit Vectors. 2023 Jun 6;16:183. doi: 10.1186/s13071-023-05802-4 (PMC10243063; doi:10.1186/s13071-023-05802-4)
Supplement: Supplementary file 2 — Additional file 2: Table S2. Hemolytic activities of recombinant defensins from body and head lice and antibiotics. [file 13071_2023_5802_MOESM2_ESM.docx]

**Table S2.** Hemolytic activities of recombinant defensins from body and head lice and antibiotics.

| **Anti-microbial peptides** | **Hemolytic activity (EC_50_, μM)** |
| --- | --- |
|  |  |
| Ampicillin | NE |
| Gentamicin | NE |
| Kanamycin | NE |
| BLDef1 | NE |
| BLDef2 | NE |
| HLDef2 | NE |

*Abbreviation: EC_50_; half-maximal effective concentration.
