# Supplementary material for: Exploring the potential role of defensins in differential vector competence of body and head lice for Bartonella quintana
Source: Parasit Vectors. 2023 Jun 6;16:183. doi: 10.1186/s13071-023-05802-4 (PMC10243063; doi:10.1186/s13071-023-05802-4)
Supplement: Supplementary file 3 — Additional file 3: Figure. S1. Amino acid sequence alignments of defensin 1 and 2 from body and head louse and other insect species. Six conserved cysteine residues are marked by red boxes. [file 13071_2023_5802_MOESM3_ESM.docx]

**
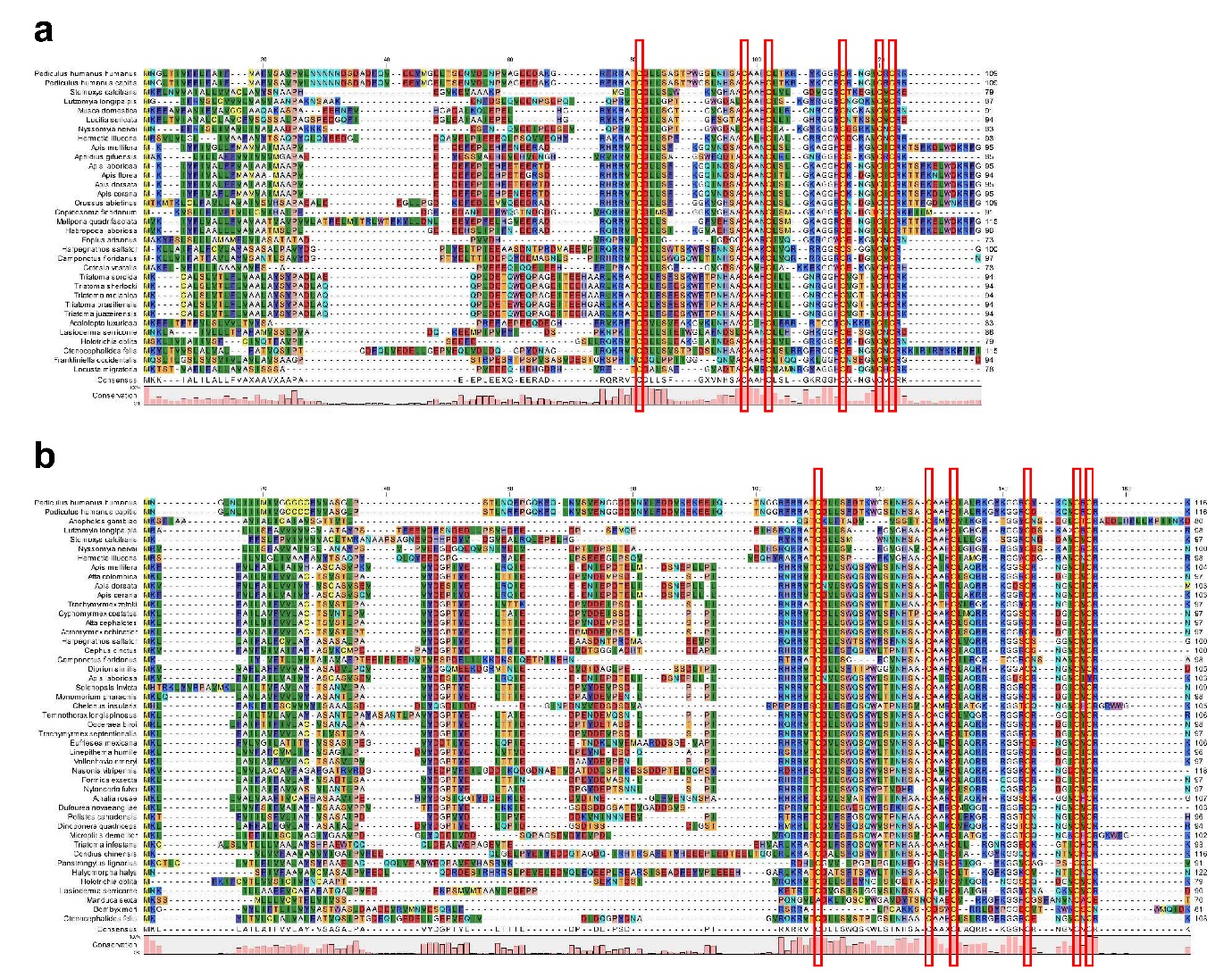
**

**Figure S1.** Amino acid sequence alignments of defensin 1 **a** and 2 **b** from body and head louse and other insect species. Six conserved cysteine residues are marked by red boxes.
